# Effects of Virgin Olive Oil and Phenol-Enriched Virgin Olive Oils on Lipoprotein Atherogenicity

**DOI:** 10.3390/nu12030601

**Published:** 2020-02-26

**Authors:** Marta Farràs, Marina Canyelles, Montserrat Fitó, Joan Carles Escolà-Gil

**Affiliations:** 1 Molecular Bases of Cardiovascular Risk Group Institut de Recerca de l’Hospital Santa Creu i Sant Pau-Institut d’Investigacions Biomèdiques (IIB) Sant Pau, 08041 Barcelona, Spain; mcanyelles@santpau.cat (M.C.); jescola@santpau.cat (J.C.E.-G.); 2 CIBER de Diabetes y Enfermedades Metabólicas Asociadas (CIBERDEM), ISCIII, 28029 Madrid, Spain; 3 Servei de Bioquímica, Hospital de la Santa Creu i Sant Pau, 08041 Barcelona, Spain; 4 Departament de Bioquímica, Biologia Molecular i Biomedicina, Universitat Autònoma de Barcelona, 08193 Barcelona, Spain; 5 Cardiovascular Risk and Nutrition Research Group, Hospital del Mar Medical Research Institute (IMIM), 08003 Barcelona, Spain; mfito@imim.es; 6 CIBER de Fisiopatología de la Obesidad y Nutrición (CIBEROBN), ISCIII, 28029 Madrid, Spain

**Keywords:** triglyceride-rich lipoprotein, chylomicrons, high density lipoprotein, low density lipoprotein, virgin olive oil

## Abstract

The atherogenicity of low-density lipoprotein (LDL) and triglyceride-rich lipoproteins (TRLs) may be more significant than LDL cholesterol levels. Clinical trials which have led to increased high-density lipoprotein (HDL) cholesterol have not always seen reductions in cardiovascular disease (CVD). Furthermore, genetic variants predisposing individuals to high HDL cholesterol are not associated with a lower risk of suffering a coronary event, and therefore HDL functionality is considered to be the most relevant aspect. Virgin olive oil (VOO) is thought to play a protective role against CVD. This review describes the effects of VOO and phenol-enriched VOOs on lipoprotein atherogenicity and HDL atheroprotective properties. The studies have demonstrated a decrease in LDL atherogenicity and an increase in the HDL-mediated macrophage cholesterol efflux capacity, HDL antioxidant activity, and HDL anti-inflammatory characteristics after various VOO interventions. Moreover, the expression of cholesterol efflux-related genes was enhanced after exposure to phenol-enriched VOOs in both post-prandial and sustained trials. Improvements in HDL antioxidant properties were also observed after VOO and phenol-enriched VOO interventions. Furthermore, some studies have demonstrated improved characteristics of TRL atherogenicity under postprandial conditions after VOO intake. Large-scale, long-term randomized clinical trials, and Mendelian analyses which assess the lipoprotein state and properties, are required to confirm these results.

## 1. Introduction

Large epidemiological studies in the 70th decade demonstrated a positive association between low-density lipoprotein (LDL) cholesterol (LDL-c) and cardiovascular disease (CVD), as well as a negative association with high-density lipoprotein (HDL) cholesterol (HDL-c) [1,2]. The association between LDL-c and CVD was first proved through a large number of randomized trials with statins, the more used cholesterol lowering drugs, and then validated by Mendelian randomization studies [3,4]. LDL is the main carrier of circulating cholesterol to the target vascular cells; hence, circulating LDL-c is a valuable indicator of the amount of lipid accumulation in the arterial wall. However, it has been found that not all LDL circulating in human blood is atherogenic; native LDL, the non-modified LDL, does not necessarily cause lipid accumulation in the arterial wall, rather it is caused by a build-up of LDL-heterogeneous modifications over time [5].

**Scheme 1:**
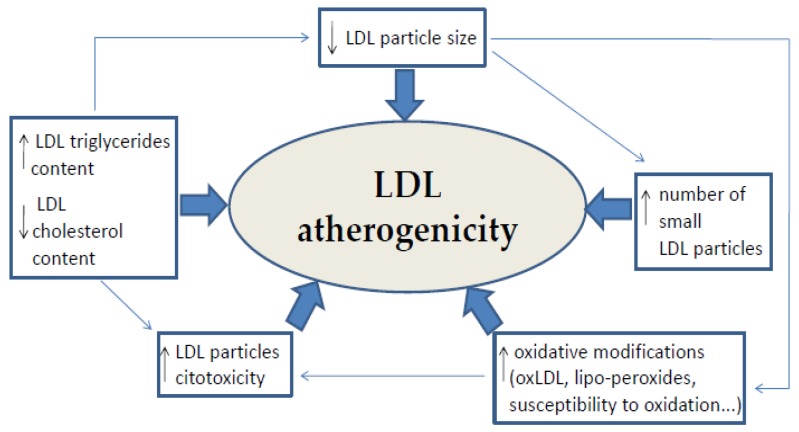
Main LDL traits associated with atherogenic processes.

The first modification in LDL particles, conferring its atherogenic properties, is the loss of acid sialic residues (desialylation) in the apolipoprotein B (apoB), which is then followed by more LDL modifications [6]. These modifications include changes in LDL composition and particle size. The increase of triglyceride (TAG) content and the decrease in all forms of cholesterol content make the LDL particle less stable; it can no longer be recognized by classical LDL receptors, but by macrophage scavenger receptors, thereby enhancing foam cell formation [7]. These changes in the lipid content determine the LDL size, causing small high densityparticles, which are strongly correlated with an atherogenic profile and incidence of cardiovascular events based on prospective study populations [8,9]. In addition, electrochemical modifications of LDL contribute toatherosclerosis. The oxidation of LDL particles, which includes heterogeneous oxidative changes in lipids and apoB, is the most studied and well established cause of atherosclerosis by LDL (Scheme 1).

Other atherogenic lipoproteins are triglyceride-rich lipoprotein (TRL), being chylomicron (CM), and very-low density lipoprotein (VLDL), the most abundant. Dietary lipid components are absorbed by enterocytes and carried in CM, and are then secreted into lymph and pass into the bloodstream. CM is exposed to lipolysis by lipoprotein lipase (LPL), which removes a significant part of their triacylglycerol (TAG) and forms smaller CM remnants, which mostly deliver the remaining TAG, cholesterol, and other lipids to the liver. CM and VLDL are too large to penetrate into the arterial intima, and only their remnants penetrate into the arterial intima and accumulate in the atherosclerotic plaque [10]. It has been demonstrated that CM remnants can cross the endothelial barrier and enter the arterial wall.

The concentration of apoB-48, the main apolipoprotein of TRL, is increased in early atherosclerosis and correlates with carotid intima-media thickness [11,12]. In studies of atherosclerotic plaque content in humans and mice, high levels of apoB-48 and apoB-100 lipoproteins have been observed, supporting the potential atherogenic role of TRLs [10,13]. One of the main mechanism involved in TRL-mediated atherogenicity is the promotion of macrophage foam cells formation, which can be exacerbated by some metabolic diseases such diabetes [14].

Another lipoprotein is HDL; HDL-c has been reported to be a strong, independent, and inverse predictor of CVD risk in many epidemiologic studies. Nevertheless, the failure of cholesteryl ester transfer proteins (CETP) inhibitors in clinical trials has generated considerable speculation about the beneficial effects of HDL [15,16]. Current data indicate that increased HDL-c levels do not always correlate with enhanced HDL functions and, therefore, should not be considered a biomarker of HDL functionality [17,18]. In addition, Mendelian randomization studies have not shown any causal effect between genetic variants that cause high HDL-c levels and the risk of myocardial infarction [19]. HDL, and mainly apoA-I, has a direct anti-inflammatory action in vascular vessels. In general terms, HDL decreases pro-inflammatory cytokine levels such as tumor necrosis factor alpha (TNFα), interleukin 6 (IL-6), and IL-8, and also inhibits the cytokine-induced expression of adhesion molecules (vascular cell adhesion molecule 1 (VCAM-1) and intercellular cell adhesion molecule 1 (ICAM-1)) [20]. 

HDL also has a double antioxidant function: it protects LDL against oxidation and attenuates the biological activity of oxLDL. The protein content of HDL determines and modulates the antioxidant action. Three of the enzymes and apolipoproteins have relevant roles: apoA-I, paraoxonase type 1 (PON1), and platelet-activating factor acetylhydrolase (PAF-AH). ApoA-I has the ability to remove oxidant LDL molecules in circulation, decrease the activity of hydroperoxides, and transport and stabilize the antioxidant enzymes (PON1 and PAF-AH) included in HDL particles [21]. PON1 is an enzyme that reduces the levels of oxidized lipids and mostly maintains the antioxidant capacity of HDL inhibiting its oxidation [22]. In mice models, PAF-AH showed an antioxidant role, but in human studies it has been correlated more with anti-inflammatory than with antioxidant activities [23]. In addition, lecithin:cholesterolacyltransferase (LCAT) may prevent the accumulation of oxidized lipids in plasma lipoproteins [24].

In addition, HDL is an important vasoprotective agent. Endothelial dysfunction, with reduced NO production, is a key factor for the perpetuation of atherosclerosis [25]. This endothelium dysfunction can be counteracted by HDL, which stimulates the release of NO and prostacyclin in endothelial cells. Consequently, NO increases vasodilatory activity [26,27] through various mechanisms such as the inhibition of platelet aggregation and adhesion, the modulation of smooth muscle cell proliferation, and the reduction of leukocyte adhesion [28]. HDL particles also have other functions, such as anti-apoptosis, cytoprotective, and anti-thrombotic [29,30]. Furthermore, HDL can protect against infections and endotoxic shock, and can improve the glucose metabolism [31,32,33].

HDL functionality can be affected in a number of ways. The accumulation of oxidized phospholipids can cause a decrease in anti-inflammatory and antioxidant functions. The action of myeloperoxidase on apoA-I can lead to dysfunctional HDL [34]. Some pathological states can also disrupt the function of HDL; the acute-phase response of inflammation causes a decrease in the levels of both HDL and apoA-I, an enrichement of TAG, and it reduces the macrophage cholesterol efflux [35]. In inflammation states, HDL loses some of these anti-inflammatory functions as a consequence of content enrichment with serum amyloid A, an acute-phase reactant during both acute and chronic inflammation [36]. HDL remodelling during inflammation also leads to a loss of some antioxidant enzymes such as PON1 and PAF-AH, causing an increased peroxidation of LDL [37].

In addition, alterations in the metabolism of HDL, concrete HDL-remodeling enzymes, and lipid transfer proteins, can affect HDL functionality. Free cholesterol is converted into cholesteryl ester within the HDL particle by the action of LCAT and generates mature αHDL, while phospholipid transferprotein (PLTP) contributes to the maintenance of HDL levels in plasma and generates nascent preβ-HDLparticles. HDL-cholesteryl ester can be transferred to apoB-containing lipoproteins by CETP and is returned to the liver through their specific receptors. Therefore, changes in the activities of LCAT, PLTP, and CETP will alter the HDL composition and subclass distribution, both of them intimately related with HDL functions.

Several studies have explored the relationship between HDL subclass distribution and CVD. A number of population studies have observed that large particles (HDL_2_) may be more cardioprotective than small ones (HDL_3_) [38,39]. Low levels of HDL_2_ and/or high levels of HDL_3_ are described in CVD [40,41,42] and diabetes [43]. Although a number of *in vitro* experiments have shown similar effects with HDL_3_ and HDL_2_,, increased circulating HDL_3_ may indicate an aberration in the maturation of HDL and in the RCT or a pro-inflammatory state of the lipoprotein [44]. In this sense, the results also suggest that HDL_2_ is more effective in promoting cholesterol efflux via SR-B1 [45,46] and ABCG-1 transporters. The HDL composition and HDL monolayer fluidity are also related to the HDL functionality. It has been observed that an HDL rich in TAG has an apoA1 prone to be unbound from HDL and with less ability to promote cholesterol efflux [47]. A lower cholesterol/phospholipids ratio is also related to a higher cholesterol efflux via the aqueous diffusion pathway [48]. Regarding HDL monolayer fluidity, a number of studies have argued that HDL with a higher fluidity is able to enhance cholesterol efflux [49].

Virgin olive oil (VOO) is the primary source of fat in the Mediterranean diet (MD). This type of diet improves the lipid profile (with an increase in HDL-c), decreases LDL oxidation, and reduces the total mortality risk [50,51,52]. In addition, our group observed that VOO increased HDL-c and decreased *in vivo* lipid oxidative damage in a dose-dependent way with olive oil phenolic compounds (OOPCs) [50]. In this context, the beneficial effects on the lipid profile of functional OOs enriched with PCs are to be expected. Taking into account these lipid profile improvements, an increase of HDL functionality and a decrease of LDL and TRL atherogenicity are also to be expected with VOO/enriched-OO consumption. The aim of this review is to report evidence of the benefits of the consumption of VOO and PC-enriched olive oil on the LDL and TRL atherogenicity as well as on HDL atheroprotective functions.

## 2. Virgin Olive Oil (VOO) and Phenol-Enriched VOOs

The Mediterranean dietary pattern is a plant-based diet rich in unsaturated fat and in various antioxidants, and lower in saturated fats. It is characterized by the consumption of VOO, nuts, plant foods, poultry, fish, moderate quantities of wine at meals, and the restriction of red and processed meat, and sweets [53]. Although strong epidemiological evidence supports the beneficial health effects of the MD, clinical trials are scarce. The PREDIMED clinical trial, a large clinical trial of the MD, demonstrated MD-mediated improvements to: a) a number of cardiovascular risk factors (classical and emerging), including: blood pressure, insulin sensitivity, lipid profile (increased HDL-c and decreased oxLDL), inflammation, oxidative stress, and carotid atherosclerosis; and b) hard-point clinical events such as the cardiovascular and total mortality risk [50,51,52].

The major compounds of OO are fatty acids (98%), and the minor compounds include sterols, alcohols, hydrocarbons, volatile compounds, and phenolic compounds (PCs) (Table 1). The principal PCs of VOO are phenolic acids, flavonoids, lignans, phenolic alcohols, and seicoroids. VOO is obtained by direct pressing or centrifugation of the olives, and it is rich in PCs (around 150-400 ppm is typical of those currently on the market).Refined OO is the OO obtained after the refining process of VOO with more acidity than 3.3 grams for every 100 grams (2.0 in the EU). In this process, some OO compounds, principally PC but also squalene, are lost [54].

In 2011, the European Food Safety Authority (EFSA) released a health claim about the beneficial effects of an intake of 5 mg/day of PC hydroxytyrosol and its related-compounds on the oxidation of LDL [55]. However, the phenolic content of the majority of OOs in the market does not achieve this quantity of PC. In this sense, phenol-enriched VOOs and functional VOO are a strategy to increase the phenolic content of VOO without increasing the amount of fat. In addition, OOs enriched with complementary phenols can obtain more beneficial effects, according to their structure/activity relationship, and can avoid the reversion of antioxidants to pro-oxidants [56,57,58].

## 3. Effects of VOO and Phenol-Enriched VOOs on LDL Atherogenicity

The LDL particle size, degree of LDL oxidative modifications, LDL resistance against oxidation, and LDL cytotoxicity appear to be the main determinants of LDL atherogenicity. The LDL particle number (LDL-P)/HDL-P ratio has the strongest independent association with CVD, permitting significant net reclassification improvements in American Heart Association/American College and Cardiology CVD risk scores [59].

A traditional MD intervention enriched with VOO was tested in humans at a high-cardiovascular risk. The diet induced a reduction of oxLDL [51], LDL resistance against oxidation, a decrease in the degree of LDL oxidative modifications, an increase in the LDL particle size, and a reduction of the LDL particles cytotoxicity. However, in the same study, a traditional MD intervention enriched with nuts did not change these LDL traits [60].

In a long-term intervention, one natural VOO, rich in PCs (366 mg/Kg), produced a decrease in plasma apoB-100 concentrations and the number of total and small LDL particles, whereas VOO with a low phenolic content increased these parameters in healthy volunteers. This VOO also increased the resistance of LDL to oxidation [61]. The same study reported a linear decrease in oxLDL in line with the phenolic content of the OO [50]. In this regard, a three-week intervention of extra VOO decreased lipo-peroxides and conjugated dienes, increased LDL susceptibility to oxidation in the lag phase, and decreased LDL susceptibility to oxidation in the maximum diene conjugated production, in comparison with a virgin argan oil intervention [62]. In this sense, 50 mL of VOO during three weeks reduced oxLDL and lipid-peroxides levels in 40 males with cardiovascular heart disease [63]. Furthermore, the long-term consumption of a polyphenol-rich water extract of olives, which was generated during OO production, also reduced oxLDL in healthy young men [64]. Another long-term intervention of functional VOO enriched with PCs from thyme and OO (FVOOT; 500 ppm) reduced the LDL-P/HDL-P ratio. A further long-term intervention with OOPC-enriched VOO (500 ppm) also decreased the number of LDL-P and particles that contained apoB-100 compared with VOO and FVOOT. After this OOPC-enriched VOO intervention, a decrease in small LDL particles was observed when compared with FVOOT, and the LDL particle size decreased when compared with VOO and FVOOT [65], indicating that a PC source could lead to differential improvements in some LDL atherogenic parameters.

In addition, in a subsample of the same study, a decrease in oxLDL was also observed after the intervention of the FVOOT [66]. In this regard, various studies have observed a direct correlation between a VOO antioxidant present in LDL and its susceptibility to oxidation, suggesting a protector effect from these antioxidants within the LDL [50,63,67].

Regarding post-prandial studies, in an acute-intake study with pre-/hypertensive patients, oxLDL improved after 30 mL of VOO and OOPC-enriched VOO (961 ppm) [68]. In addition, a decrease in oxLDL was observed after an acute ingestion of phenol-enriched OO (25 g) [69]. Nevertheless, a single dose of VOO (50 mL) increased the oxLDL and lipid-peroxides at 6h post-prandial, in healthy volunteers [70]. In this respect, regarding functional OO, in a post-prandial study with healthy individuals, oxLDL increased after VOO interventions (30 mL) with low- (250 ppm) and high-phenolic (750 ppm) content. Therefore, the quantity ofPCsis a key factor regarding the improvement of the LDL oxidative state under post-prandial conditions in healthy humans. This could be because high doses of antioxidants might revert to pro-oxidants [56,57,58]. In this regard, VOO enriched with complementary phenols (according to their structure/activity relationship) could be a potential option to obtain more healthy effects.

In Table 2, we summarize the clinical trials which analysed the effects of VOO and phenol-enriched VOO on LDL atherogenicity. This research field deserves further investigation.

The three large long-term studies (*n* = 930, *n* = 210, and *n* = 200) demonstrated the decrease of LDL atherogenicity within and outsidethe context of a Mediterranean diet. In fact, in the Eurolive Study [50], the improvement of the LDL oxidation was achieved in a multicentre-project conducted in five European countries. In all the studies, a similar amount of VOO was employed, 25–30 mL of raw VOO/day or 1 L/week of total VOO for the family unit (50 mL of OO for raw and cooking purposes, approximately). In concordance with large studies, the other studies with smaller sample sizes observed similar results.

## 4. Effects of VOO and Phenol-Enriched OO on Post-Prandial Lipemia

Post-prandial trygliceridemia is a potential independent cardiovascular risk factor [72,73,74]. A postprandial accumulation of TRL indicates greater atherogenicity. Indeed, a delayed clearance of post-prandial TRL has been observed in humans with atherosclerosis [75]. A potential mechanism to explain this is that delayed clearance could give more time for the LPL actionand therefore enhance the production of small CMs that could be retained in the artery wall, enhancing their atherogenicity.

MD rich in OO improve post-prandial lipemia and remnant cholesterol concentrations primarily in patients with type 2 diabetes when compared with a low-fat diet in a large study with 557 subjects [76]. Several reports have evaluated the potential of OO to modulate post-prandial TAG accumulation, generally measured with the area under curve (AUC). Indeed, AUCs of plasma TAG and CMs rich in TAG were higher after a butter meal compared to an OO one in type 2 diabetes patients [77]. In contrast, another study shows that meals containing butter reduce post-prandial lipemia and CM accumulation in the circulation better when compared with meals containing OO or sunflower oil in healthy young men [78], indicating that OO health benefits may be more important in type 2 diabetic patients, possibly because of higher post-prandial lipemia. In this regard, a trend in the reduction of TG AUC was also observed after a high monounsaturated (MUFA) diet (supplemented with VOO) compared to a low fat diet in type 2 diabetes mellitus. However, no differences were observed between the two diets [79].

Interestingly, TAG AUC was higher after the consumption of high oleic sunflower oil in comparison to VOO in normolipemic subjects [80]. Another study also demonstrated that TAG AUC was higher after the consumption of high oleic sunflower oil in comparison to VOO or enriched-VOO in normolipemic subjects. However, no differences were observed between the two VOOs, suggesting that the unsaponifiable fraction does not have any effect on post-prandrial trygliceridemia [81]. In line with these findings, the consumption of pomace olive oil (POO), which is a sub-product of VOO elaboration and which is rich in PCs, did not show any difference from VOO in the TAG AUC [82]. 

Regarding the TAG composition of TRL particles and their size, large TAG-rich CMs are preferentially hydrolyzed by lipoprotein lipase and then rapidly converted into CM remnants. The fatty acid composition of CMs is an important determinant of CM size and, consequently, its metabolism. Polyunsaturated fatty acids (PUFAs) increase the CM size when compared to saturated fatty acids (SFAs) in humans [83,84]. Studies in experimental models have demonstrated that CMs enriched with n-6 PUFAs can be hydrolyzed more rapidly by lipoprotein lipase than CMs enriched with SFAs, MUFAs, and *n*-3 PUFAs [85]. Human data have shown that CM contains more TAGs two to four hours after OO (rich in MUFA) and sunflower oil (rich in *n*-6 PUFA) meals than after butter (rich in SFAs) intake, and that a butter meal induced higher VLDL TAGs than OO or sunflower oil meals in healthy young men [78]. Nevertheless, in post-menopausal women, the consumption of OO resulted in higher concentrations of apoB-48 and a lower TAG/apoB-48 ratio compared to other oils rich in SFAs and PUFAs (fish oil, safflower oil, and a mix of fish and safflower oils). Moreover, a tendency to increase retinyl ester in TRLs was observed after OO consumption [86]. These data indicate that OO MUFAs increase the production of CMs and that they are highly hydrolyzed by lipoprotein lipase. In this context, in overweight patients with type 2 diabetes, a meal supplemented with 80g of OO presented lower levels of plasma TAGs and TAGs in CMs compared to a meal supplemented with butter, indicating that OO could have a positive impact on patients diagnosed with type 2 diabetes [77]. 

It should be noted that after a VOO meal, CM phospholipids were enriched with oleic acid and n-3 PUFA, but with stearic and linoleic acids after a high-oleic sunflower oil meal [87]. This could indicate that the fatty acid composition of phospholipids can be important for the clearance of TRL in post-prandial conditions. The higher oleic acid levels in the phospholipids of TRL and the lower levels of stearic acid could explain the enhanced hydrolysis of phospholipids and probably the enhanced hydrolysis of VOO TRLs. Therefore, VOO-mediated changes to the composition of phospholipids in TRL may influence lipolysis and TRL uptake by hepatic receptors [87]. Importantly, in healthy young men, POO produced a lower number of TRL particles with a higher TAG concentration and a higher particle size in comparison with refined OO. In addition, after the consumption of POO, particles were rapidly depleted of triglycerides. These data suggest that OOPC could activate the hydrolysis of TAG by LPL [82]. 

A summary of the clinical trials which analysed the effects of OO, VOO, and phenol-enriched VOOs on post-prandial lipemia is shown in Table 3. The majority of these studies have a reduced sample size (from 8 to 12 subjects), indicating a need for more large studies in this field. Three clinical trials study the effects of natural VOO, while only one assessed the effects of phenol-enriched VOO. The ingestion of these VOOs promoted in general a decrease of TAG AUC.

## 5. Effects of VOO and Phenol-Enriched Olive Oils on TRLs Atherogenicity

A critical step in the process of circulating TRL removal is the hepatic uptake. As a result, several reports have evaluated the effects of fatty acid intake from different sources on TRL uptake by culture hepatocytes. Indeed, findings from experimental models clearly demonstrate that CM remnant binding and internalization are influenced by TRL fatty acid composition. However, only TRL particles enriched with 3-PUFA (derived from fish oil) appear to be taken up faster than those enriched with saturated fat (derived from palm oil), MUFA (derived from olive oil), or 6-PUFA (corn oil) [88]. Furthermore, the hepatocyte uptake of TRLs derived from human serum after the intake of meals with enriched-VOO was similar to that of VOO. Enriched-VOO affected the molecular species incorporated into chylomicrons in the intestine, but the unsaponifiable fraction of VOO did not affect the hepatic TRL uptake [89].

It should be noted that TRL-like particles enriched in saturated fatty acids or n-6 PUFAs (derived from palm and corn oil) up-regulate the hepatocyte VLDL secretion, whereas those enriched in MUFAs (derived from OO) were unaffected, indicating that MUFAs from TRLs have minor effects on VLDL secretion when they reach the liver [90]. Increasing the unsaponifiable content of VOO did not exert any further effects on lipid accumulation in primary hepatocytes or on the VLDL secretion when compared with the effects of VOO [89].

Previous reports have demonstrated that SFA-enriched meals (butter) usually enhance post-prandial lipid peroxide levels and the inflammatory status of peripheral blood mononuclear cells in the postprandial state compared with meals enriched with MUFA from OO [91,92]. Interestingly, when the potential of VOO and its natural antioxidants on the post-prandial inflammatory response was evaluated in obese subjects, both reduced NF-kB activation and increased IkBα in peripheral blood mononuclear cells, while also reducing the LPS plasma concentration [93].

Minor components from OO may also form part of TRL, thereby permitting interaction with the cells implicated in endothelial dysfunction and atherogenesis. The potential of VOO to alter the post-prandial TRL interaction with human vascular cells was investigated by determining the effects of VOO and high-oleic sunflower oil on the ability of TRL to interact with umbilical vein endothelial and aorta smooth muscle cells [94]. This report provided evidence of the interaction of VOO TRL with human vascular cells. In this context, the effects of the unsaponifiable fraction of VOO on the ability of TRL to modify the production of NO and vasoactive eicosanoids by human endothelial cells were also investigated [81]. TRLs derived from human serum after the intake of meals enriched in high-oleic sunflower oil, VOO, or enriched VOO did not differ in NO release, but enriched VOO TRLs reduced the production of pro-inflammatory and pro-thrombotic substances that play important roles in the dysregulation of vascular reactivity, such as PGE2 and TxB2, when compared with the other dietary oils. However, the authors could not conclude which components of the unsaponifiable fraction of VOO were involved in these effects.

Fatty acid composition may also affect the macrophage TRL uptake and induction of lipid accumulation in macrophage foam cells. SFA- or MUFA-enriched TRL-like particles were taken up more rapidly than those enriched in 3-or 6-PUFAS, and this change resulted in an enhanced lipid accumulation in the case of SFAs, but not for MUFA-rich TRL particles [95]. It should be noted that the exposure of human macrophage THP-1 cells to TRL-like particles enriched with fat from different VOOs induced intracellular lipid accumulation. In addition, linoleic acid-rich extra VOO enhanced lipid incorporation into THP-1 macrophages when compared to that induced by oleic acid-rich VOO [96]. However, the effects of VOO PCs remain unclear.

## 6. Effects of VOO and Phenol-Enriched VOOs on HDL Characteristics and Metabolism

VOO consumption induces changes in the HDL composition. Indeed, VOO consumption in humans produces a trygliceride-poor core [49] and a rise in the levels of apo-A1 [97] and apoA-IV [98]. This apoA-IV increment has also been reported in hypercholesterolemic apoE-deficient mice after a VOO-rich diet [99]. An increase of apo-A1 was observed after an MD supplemented with VOO when compared to MD supplemented with nuts [100]. In contrast, a decrease of apo-A1 after an MD has also been describedwhen compared to a saturated diet [101]. In this regard, it could indicate aparallel increase in the HDL particle count after the Mediterranean diet. Studies have shown that VOO improves the HDL monolayer fluidity in healthy humans [49,102]. In this regard, VOO interventions also modify the HDL subclass distribution and particle numbers. It has been reported that a three-weeks consumption of VOO in healthy individuals induces an increasing trend in HDL particle numbers [49]. In addition, a VOO [49] and MD with VOO [103] were observed to increase large HDL particles (HDL_2_) in humans. Similar results were also found in rats after supplementation with OOPCs [104]. Long-term VOO consumption also decreased small HDL particles (HDL_3_) and was able to incorporate OO metabolites into HDL in healthy humans [49]. A FVOOT (500 ppm) also increased levels of HDL_2_ and decreased HDL_3_ in hypercholesterolemic volunteers. It also improved the HDL composition, increasing the esterified cholesterol/free cholesterol and phospholipids/free cholesterol ratios in HDL [105]. Taken together, these findings indicate that VOO and phenol-enriched VOOs improved the HDL size and composition.

A number of studies have reported the effects of VOO on the main HDL-remodeling enzymes and lipid transfer proteins. CETP and LCAT did not change after a sustained VOO intervention in healthy humans [49]. Similarly, a sustained OO intervention of 60 ml/day in males with mild hypertension did not change these enzymes [106]. In line with these findings, dietary oleic acid had no effects on LCAT versus corn oil in hamsters [107]. Again, a nine-weeks intervention of a cholesterol-enriched diet with OO (200 g/kg) in hamsters showed no differences in LCAT, CETP, or PLTP [108]. After a post-prandial intervention of VOO (7 mL) in rats, PLTP hepatic gene expression increased, although LCAT gene expression did not change [109]. In contrast, squalene (1 g/kg) raised the LCAT hepatic expression in apoa1- and apoe-deficient mice [110]. Overall, these findings indicate that VOO and their PCs may alter the HDL size by increasing the number of large HDL particles. However, this effect seems associated with the apoA-1 composition rather than with any effects on the main HDL remodeling enzymes and lipid transfer protein activities. 

## 7. Effects of VOO and Phenol-Enriched VOOs on HDL-Mediated Cholesterol Efflux Capacity

Macrophage cholesterol efflux capacity is considered the main clinically atheroprotective property of HDL. An altered cholesterol efflux could reflect the presence of subclinical CVD better than HDL-c levels [111,112].

Our group showed for the first time and provided the first level of evidence that the intake of a real-life VOO dose (366mg/kg) improved cholesterol efflux in humans [49]. Our group also demonstrated that FVOOT (500 ppm) increased cholesterol efflux compared with an OOPC-enriched VOO (500 ppm) in hypercholesterolemic patients. However, this increment was not observed when compared to the baseline or to a natural VOO. It was also observed that HDL fluidity, ApoA-I, and the oxidative status are the principal determinants for cholesterol efflux after consumption periods independently of the kind of VOO consumed [113].

An intervention with a traditional MD, in the frame of the PREDIMED study, also increased cholesterol efflux in humans at cardiovascular risk [114]. Specifically, the augmentation of the intake of VOO, whole grain, and fish (achievable through a regular diet) were associated with improvements in the HDL cholesterol efflux capacity in subjects at high cardiovascular risk [115]. PCs and fatty acids are potent ligands of the PPARs family and other nuclear factors involved in RCT. Additionally, in this regard, few studies have analyzed the direct effect of VOO on the cholesterol efflux-related gene expression profile. Hydroxytyrosol, the main PC from OO, has been demonstrated to enhance the peroxisome proliferator-activated receptors (*PPARα* and *PPARγ*) of gene expression in 3T3-L1 adipocytes [116]. In humans, a post-prandrial VOO intervention of 50 mL augmented the expression of *MED1* and *CD36* in peripheral blood mononuclear cells [70]. The ATP-binding cassette transporter A1 (*ABCA1*) and *ABCG1* increased in a long-term non-controlled clinical trial with VOO in healthy individuals, whereas this intervention reduced the expression of *SCARB1* in human macrophages [102]. However, a long-term diet intervention rich in VOO did not show any enhancement of *ABCA1* in peripheral blood mononuclear cells in healthy volunteers [117].

An increase of cholesterol efflux-related gene expression in peripheral blood mononuclear cells was observed after an intervention with a FVOOT (500 ppm) in hypercholesterolemic individuals; concretely, FVOOT upregulated *CYP27A1*, *CAV1*, *LXRβ, RXRα,* and *PPARβ/δ.* In addition, gene expression in the white blood cells of the cholesterol efflux transporter genes *ABCA1*, scavenger receptor B1 (*SCARB1*), and some transcription factors related to the peroxisome proliferator-activated receptors (*PPARα*, *PPARγ*, *PPARβ/δ*, and *MED1*), also increased after an acute intervention of an OOPC-enriched VOO (961 mg/kg) in pre-/hypertensive patients when compared with a control VOO (289 mg/kg) [68]. Taken together, these findings indicate that VOO and phenol-enriched VOOs can up-regulate a number of genes involved in regulating macrophage cholesterol efflux.

## 8. Effects of VOO and Phenol-Enriched VOOs on HDL Antioxidant Activity 

The potential of VOO to improve the ability of HDL particles to protect against LDL oxidation has been investigated in several reports. This capacity is important because oxLDLs are a key trigger for the onset of atherosclerotic plaque [118]. 

VOO (concretely, the OOPC) intake has been dose-dependently associated with a reduction in oxLDLs *in vivo* [50]. Part of this protection could be produced for an induction of the HDL antioxidant function. Indeed, it has been reported that a VOO-rich diet increased HDL antioxidant activity in hypercholesterolemic apoE-deficient mice with spontaneous atherosclerosis development [118]. In addition, a VOO-rich intervention augmented the main PON1 antioxidant activity in healthy men [62]. A FVOOT consumption also augmented the PON1 activity in hypercholesterolemic individuals [105]. In this context, the LCAT mass was found to be increased after this intervention in hypercholesterolemic individuals, but the PAFH activity did not show any increment after VOO or phenol-enriched VOOs [105]. It should be noted that VOO intake was able to improve the apoA-I functionality in HDL lipoproteins, preventing protein oxidation and leading to a more stable conformation of apoA-I in HDLs [49]. These characteristics may also improve HDLs’ capacity to pick up oxidized lipids [119].

## 9. Effects of VOO and Phenol-Enriched VOOs on HDL Anti-Inflammatory Capacity

HDL lipoproteins have anti-inflammatory activities which could reduce the chronic inflammatory response in the arterial wall, avoiding later LDL accumulation [120]. HDL particles also reduce monocyte enhancement and adhesion in the endothelium [121], decrease neutrophil activation [122], and also decrease neutrophil signaling via toll-like receptors [123]. VOO consumption has been shown to be highly protective for vascular response and endothelial integrity, as observed in a number of VOO-rich interventions in humans [124,125,126].

The consumption of VOO appears to improve the HDL anti-inflammatory capacity, mainly by increasing the ability of HDLs to block the secretion of ICAM-1 and the adhesion of monocytes to endothelial cells in healthy humans [127]. As reported above, the potential of VOO in regulating PON-1 activity could also improve its anti-inflammatory properties [127]. Inflammatory states increase the content of the acute phase bound to HDLs, transforming the lipoproteins into pro-inflammatory and dysfunctional ones [119]. It has been hypothesized that an antioxidant-rich intervention, such as a VOO one, could decrease the acute-phase protein levels onto the lipoprotein. The consumption of different VOOs [98] and a VOO rich MD [128] could also reduce the content of acute-phase proteins in HDLs, enhancing a less pro-inflammatory lipoprotein status.

## 10. Effects of VOO and Phenol-Enriched VOOs on HDL Vasoprotective Capacity

VOO consumption has been shown to be highly protective for vascular response and endothelial integrity, as observed in a number of VOO-rich interventions in humans [124,125,126]. HDLs can act as transporters of several derivatives of OOPCs to the endothelial cells where they may prevent oxidative damage in cell mitochondria and preserve the production of NO, as reported in *in vitro* studies [125,129]. In addition, in healthy individuals, an OOPC intervention activated the HDL capacity to reduce ICAM-1 secretion and monocyte adhesion to endothelial cells [127]. 

There are no studies reporting the effects of phenol-enriched VOO on the HDL-mediated vasoprotective function. Nevertheless, the acute consumption of an OOPC-enriched VOO *in vivo* in pre-/hypertensive humans improved the endothelial function, measured as ischemic reactive hyperemia (IRH) [124]. In line with these findings, an acute consumption of functional VOO enriched with its own PCs (500 ppm and 750 ppm) *in vivo* in healthy humans improved the IRH. In addition, a sustained intake of functional VOOs enriched with their own PC or combined with thyme PC also improved IRH in hypercholesterolemic patients [71].

In addition, systemic biomarkers related to the endothelial and vasoprotective functions have been studied after VOO and functional VOO interventions. The plasminogen activator inhibitor type 1 (PAI-1) and high sensitive C-reactive protein (hsCRP) improved after an acute intervention with VOO and with high-phenolic content VOO in pre-/hypertensive individuals. In these individuals, VCAM-1 was also reduced after a high-phenolic content VOO [124]. PAI-1 was also found to decrease after an acute intervention of low-, medium-, and high-phenolic content VOOs. Endothelin-1 increased and NO decreased after an acute consumption of low- and high-phenolic content VOO. However, a sustained study with phenol-enriched VOOs did not show significant changes in these parameters [71].

Table 4 shows the main clinical trials which analysed the effects of VOO and phenol-enriched VOO on HDL characteristics and functions reported in Section 6, Section 7, Section 8, Section 9 and Section 10. The largest studies (*n* = 551, *n* = 296, *n* = 169) tested VOO in the context of a Mediterranean diet and demonstrated increased HDL_2_, HDL ability to sterify cholesterol, HDL cholesterol efflux capacity, and HDL antioxidant and vasodilatory activities after VOO consumption in high-cardiovascular risk patients. Changes in HDL composition, HDL monolayer fluidity, and HDL cholesterol efflux-related gene expression were only demonstrated in clinical trials with less sample size (from 13 to 60 subjects). A similar amount of VOO, 25–30 mL of raw VOO/day or 1 L/week of total VOO for the family unit (50 mL of OO for raw and cooking purpose approximately), was used in all the studies described. The enrichment of VOO with OOPC or with complementary antioxidants (OOPC and thyme PC) showed more beneficial effects on HDL function than VOO in hypercholesterolemic and pre-/hypertensive patients, indicating a potential tool for these types of patients. There are no studies assessing the HDL functions on post-prandial state after the consumption of VOO or phenol-enriched VOO, only cholesterol efflux-related gene expression was tested in post-prandial state.

## 11. Conclusions

Phenol-enriched VOO is a strategy to increase VOO phenoliccontent without increasing its fat content and, therefore, enhances the potential of VOO to improve lipoprotein functions. VOO reduces oxLDL levels in long-term studies, while enhancing its resistance to oxidation. The improvement in lipo-peroxidation is closely associated with the OO phenolic content. VOO enriched with OOPC also reduces the number of LDL-P and atherogenic small LDL particles. OO improves postprandial lipemia, but only in patients with type 2 diabetes; however, OOPCs do not appear to regulate postprandial TAG response. In contrast, VOO and phenol-enriched OO may counteract several atherogenic characteristics of TRL under postprandial conditions, such as its ability to stimulate the production of pro-inflammatory and pro-thrombotic substances and its accumulation in macrophage foam cells. VOO and phenol-enriched VOO also enhance several HDL-mediated atheroprotective functions, including the ability to stimulate macrophage cholesterol efflux and HDL antioxidant and anti-inflammatory properties. Some of these changes are related to dietary VOO effects on HDL size, the up-regulation of several transporters involved in cholesterol efflux, and the increase in HDL antioxidant enzymes. 

Strong evidence supports the positive effects of VOO and phenol-enriched VOO in counteracting lipoprotein atherogenicity. Nevertheless, more large-scale and long-term randomized clinical trials to assess the effects of VOO (within the frame of usual diet) on cardiovascular complications and mortality are required, in different populations and individuals with different pathologies.

## Figures and Tables

**Table 1:** Olive oil composition.

SAPONIFIABLE FRACTION (about 98%)	UNSAPONIFIABLE FRACTION (about 2%)
Monounsaturated fatty acids (55%–83%): oleic acid Polyunsaturated fatty acids (11%–42.5%): linolenic acid, palmitoleic acid, linoleic acid Saturated fatty acids (8%–25.1%): palmitic acid, myristic acid, stearic acid	Lipophilic phenolics: tocopherols and tocotrienols Hydrophilic phenolics: phenolic acids, phenolic alcohols, seicoroids, lignans, and flavones Volatile compounds Pigments: clorophylls Hydrocarbons: squalene, β-carotene, lycopene Sterols: β-sitosterol, campesterol, estigmasterol Triterpene and aliphatic alcohols Non-glyceride esters: alcoholic and sterol compounds, waxes

**Table 2:** Clinical trials which analysed the effects of VOO and phenol-enriched VOO on LDL atherogenicity.

Interventions	Duration	Trial Design	Participants (*n* and Health Status)	Doses	OO Effects	Reference
- MD + VOO - MD + nuts - Low-fat control diet	1 year	Parallel Randomized Controlled	930, subjects	1 L/week of VOO 210 g/week of nuts	MD+VOO: ↓ oxLDL	[51]
- MD + VOO - MD + nuts - Low-fat control diet	1 year	Parallel Randomized Controlled	210, high-cardiovascular risk patients	1 L/week of VOO 210 g/week of nuts	MD+VOO: ↑ LDL resistance against oxidation ↓ degree of LDL oxidative modifications ↑ LDL particle size ↑ LDL-cholesterol rich particles ↓ LDL particles cytotoxicity	[60]
- Low-PC VOO (2.7 mg/kg) - Medium-PC VOO (164 mg/kg) - High-PC VOO (366 mg/kg)	3 weeks	Cross-over Randomized Controlled	200, healthy men	25 mL/d	Low-PC VOO > Medium-PC VOO > High-PC VOO: ↓ oxLDL	[50]
- Virgin argan oil - Extra VOO	21 days	Parallel Randomized Controlled	60, healthy young men	25 mL/d	Extra-VOO: ↓ Lipo-peroxides and conjugated dienes ↑ LDL susceptibility to oxidation in the lag phase ↓ LDL susceptibility to oxidation in the maximum diene conjugated production	[62]
- ROO (14.7 mg/Kg) - VOO (161 mg/Kg)	3 weeks	Cross-over Randomized Controlled	40, males with stable cardiovascular heart disease	50 mL	VOO: ↓ oxLDL ↓ lipo-peroxides	[63]
- Polyphenol-rich water extract microencapsulated	4 weeks	-	35, healthy young men	30 g	PC-rich water extract: ↓ oxLDL	[64]
- VOO (80 ppm) - OOPC-enriched VOO (500 ppm) - FVOOT (500 ppm)	3 weeks	Crossover Randomized Double-blind Controlled	33, hypercholesterolemic volunteers	25 mL/d	FVOOT: ↓ LDL-P/HDL-P ↓ oxLDL (in a subsample) OOPC-enriched VOO: ↓ LDL-P ↓ apoB100-containing lipoproteins ↓ small LDL particles ↓ LDL particle size	[65,66]
- Low-PC VOO (2.7 mg/kg) - High-PC VOO (366 mg/kg)	3 weeks	Cross-over Randomized Controlled	25, healthy men	25 mL/d	High-PC VOO: ↓ plasma apoB-100 ↓ total LDL-P and small LDL-P ↑ resistance of LDL to being oxidized	[61]
- Phenol-enriched extra VOO - Control oil	Acute ingestion	Randomized Controlled	22, healthy volunteers	25 g	PC-enriched extra VOO: ↓ oxLDL	[69]
- Moderate OOPC-enriched VOO (289 mg/kg) - High OOPC-enriched VOO (961 mg/kg)	Acute ingestion	Crossover Randomized Double-blind Controlled	13, pre-/hypertensive patients	30 mL	Moderate OOPC-enriched VOO: ↓ oxLDL High OOPC-enriched VOO: ↓ oxLDL	[68]
- Low OOPC-enriched VOO (250 ppm) - Medium OOPC-enriched VOO (500 ppm) - High OOPC-enriched VOO (750 ppm)	Acute ingestion	Crossover Randomized Double-blind Controlled	12, healthy subjects	30 mL	Low OOPC-enriched VOO: ↑ oxLDL High OOPC-enriched FVOO: ↑ oxLDL	[71]
- VOO	Acute ingestion	-	11, healthy volunteers	50 mL (44g)	VOO: ↑ oxLDL ↑ lipid peroxides	[70]

apo: apolipoprotein; AUC: area under curve; CH: carbohydrate; CM: chylomicrons; MUFA: monounsaturated fatty acids; OO: olive oil; P: number of particles; POO: pomace olive oil; PUFA: polyunsaturated fatty acids; ROO: refined olive oil; TAG: tryglicerides; TRL: trygliceride-rich lipoprotein; VOO: virgin olive oil.

**Table 3:** Clinical trials which analysed the effects of OO, VOO and phenol-enriched VOO on post-prandial lipemia.

Interventions	Duration	Trial Design	Participants (*n* and Health Status	Dose	OO Effects	Reference
- MD + OO - Low fat diet	3 year	Parallel Randomized Controlled	557, type 2 diabetes mellitus patients	-	OO: ↓ post-prandial lipemia ↓ remnant cholesterol	[76]
- Control meal - Control meal + butter - Control meal + OO	Post-prandial	Cross-over Randomized Controlled	12, type 2 diabetes and overweight patients	100 g of butter 80 g of OO	OO: ↓ plasma TAG ↓ TAG in CM	[77]
- High CH diet - High MUFA diet (with OO)	6 weeks	Cross-over Randomized Controlled	12, type 2 diabetes mellitus	High CH diet: 16.3 ± 7.6 g OO/d High MUFA diet: 55.6 ±4.2 g OO/d	OO: ↓ TAG AUC	[79]
- Test meal + high oleic sunflower oil - Test meal + VOO - Test meal + enriched VOO (oils were mixed with tomato sauce)	Post-prandial	Cross-over Controlled	10, normolipidemic subjects	70 g of OOs	VOO: ↓ TAG AUC Enriched-VOO: ↓ TAG AUC	[81]
- Control meal - Control meal + butter - Control meal + sunflower oil - Control meal + OO	Post-prandial	Randomized Cross-over Controlled	10, healthy men	40 g of oils 40 g of butter	OO: ↑ TAG in CM (2–4h after intervention) ↓ TAG in VLDL	[78]
- Test meal + palm oil - Test meal + safflower oil - Test meal + mix of fish and safflower oils - Test meal + OO	Post-prandial	Single-blind Randomized Cross-over Controlled	10, Post-menopausal women	40 g of oils	OO: ↑ apoB-48 ↓ TAG/apoB-48	[86]
- Test meal + ROO - Test meal + POO (oils were mixed with tomato sauce)	1 week	Cross-over Randomized Controlled	9, healthy young men	70 g of oils	POO: No differences TAG AUC ↓ TRL particles ↑ TRL TAG after 2h intake ↓ TRL TAG after 4h intake ↑ particle size ↑ rapid depletion of TAG in particles	[82]
- Control meal - Control meal + VOO - Control meal + high oleic sunflower oil (oils were mixed with tomato sauce)	Post-prandial	Randomized Cross-over Double-blind Controlled	8, normolipidemic men	70 g of OOs	VOO ↓ TAG TRL AUC	[80]
- Control meal - Control meal + VOO - Control meal + high oleic sunflower oil (oils were mixed with tomato sauce)	Post-prandial	Randomized Controlled Cross-over	8, normolipidemic men	70 g of oils	VOO: ↑ oleic acid and *n*-3 PUFA in CM phospholipid	[87]

apo: apolipoprotein; AUC: area under curve; CH: carbohydrate; CM: chylomicrons; MUFA: monounsaturated fatty acids; OO: olive oil; POO: pomace olive oil; PUFA: polyunsaturated fatty acids; ROO: refined olive oil; TAG: tryglicerides; TRL: trygliceride-rich lipoprotein; VOO: virgin olive oil.

**Table 4:** Clinical trials which analysed the effects of VOO and phenol-enriched VOO on HDL characteristics and functions.

Interventions	Duration	Trial Design	Participants (*n* and Health Status)	Dose	OO Effects	Reference
- MD + VOO - MD + nuts - Low-fat control diet	3 months	Parallel Randomized Controlled	551, highcardiovascular risk individuals	1 L/week of VOO 210 g/week of nuts	MD + VOO: ↑ apo-A1	[100]
- Control diet - MD + nuts - MD + VOO	1 year	Parallel Randomized Controlled	296, cardiovascular risk patients	1 L/week of VOO 30g/day of nuts	MD+VOO: ↑ cholesterol efflux ↓ CETP ↑ HDL ability to esterify cholesterol ↑ PON-1 arylesterase activity ↑ HDL vasodilatory capacity	[114]
- Control diet - MD + nuts - MD + VOO	1 year	Parallel Randomized Controlled	169, highcardiovascular risk patients	1 L/week of VOO 30g/day of nuts	MD + VOO: ↑ HDL_2_	[103]
- Virgin argan oil - Extra VOO	21 days	Parallel Randomized Controlled	60, healthy young men	25 mL/d	Extra-VOO: ↑ PON-1 activities	[62]
- Extra VOO - Argan oil	3 weeks	Randomized Controlled	60, healthy volunteers	25 mL/d	Extra VOO: ↑ apo-A1	[97]
- Low-PC VOO (2.7 mg/kg) - High-PC VOO (366 mg/kg)	21 days	Cross-over Randomized Controlled	47, healthy men	25 mL/d (22g)	High-PC VOO: ↓ TAG in HDL ↑ ApoA1 in HDL ↑ HDL monolayer fluidity ↓ HDL_3_ ↑ OO metabolites in HDL ↑ cholesterol efflux	[49]
- Saturated fat-rich diet - Low-fat diet - MD diet + VOO	4 weeks	Controlled Cross-over	41, normolipidemic men	-	MD + VOO: ↓ apo-A1	[101]
- VOO (80 ppm) - OOPC-enriched VOO (500 ppm) - FVOOT (500 ppm)	3 weeks	Cross-over Randomized Double-blind Controlled	33, hypercholesterolemic volunteers	25 mL/d	FVOOT: ↑ apoA-IV ↑ PON1 activity ↑ HDL_2_ ↓ HDL_3_ ↑ HDL esterified/free cholesterol ↑ HDL phospholipids/free cholesterol ↑ LCAT ↑ cholesterol efflux ↓ acute-phase proteins in HDL ↓ total LDL-P/total HDL-P small ↓ HDL/large HDL ↓ HDL-cholesterol/HDL-P ↑ cholesterol efflux-related genes: *CYP27A1*, *CAV1*, *LXRβ, RXRα,* and *PPARβ/δ*	[65]
- Extra-VOO	12 weeks	Non-controlled trial	26, healthy volunteers	25 mL/d	Extra-VOO: ↑ HDL monolayer fluidity ↑ cholesterol efflux	[102]
- Extra-VOO	12 weeks	Non-controlled trial	20, heathy normolipemic non-smokers volunteers	25 mL/d	Extra-VOO: ↑ HDL capacity to reduce ICAM-1 secretion and monocyte adhesion to endothelial cells ↑ anti-inflammatory activity of PON1	[127]
- Moderate OOPC-enriched VOO (289 mg/kg) - High OOPC-enriched VOO (961 mg/kg)	Post-prandial	Cross-over Randomized Double-blind Controlled	13, pre-/hypertensive patients	30 mL	High OOPC-enriched VOO: ↑ cholesterol efflux-related genes: *ABCA1, SCARB1, PPARα*, *PPARγ*, *PPARβ/δ*, and *MED1*	[68]

ABCA1: ATP-binding cassette transporter A1; ApoA1: apolipoprotein A1; CAV1: caveolin 1;CETP: cholesteryl ester transfer protein; CYP27A1: cytochrome P450 family 27 subfamily A member 1; ICAM-1: intercellular adhesion molecule 1; LCAT: lecithin:cholesterol acyltransferase; LXRβ: liver X receptor beta; MED1: peroxisome proliferator-activated receptor binding protein; MD: mediterranean diet; OO: olive oil; P: number of particles; PC: phenolic compounds; PPARα: peroxisome proliferator-activated receptor alpha;PPARβ/δ: peroxisome proliferator-activated receptor beta/delta; PPARγ: peroxisome proliferator-activated receptor gamma; PON: paraoxonase; RXRα: retinoid X receptor alpha, SCARB1: scavenger receptor class B member 1; TAG: tryglicerides; VOO: virgin olive oil.

## References

[1] Castelli W.P., Doyle J.T., Gordon T., Hames C.G., Hjortland M.C., Hulley S.B., Kagan A., Zukel W.J. (1977). HDL cholesterol and other lipids in coronary heart disease. The cooperative lipoprotein phenotyping study. Circulation.

[2] Castelli W.P., Garrison R.J., Wilson P.W., Abbott R.D., Kalousdian S., Kannel W.B. (1986). Incidence of coronary heart disease and lipoprotein cholesterol levels. The Framingham Study. JAMA.

[3] Kearney P.M., Blackwell L., Collins R., Keech A., Simes J., Peto R., Armitage J., Baigent C. (2008). Efficacy of cholesterol-lowering therapy in 18,686 people with diabetes in 14 randomised trials of statins: A meta-analysis. Lancet.

[4] Linsel-Nitschke P., Gotz A., Erdmann J., Braenne I., Braund P., Hengstenberg C., Stark K., Fischer M., Schreiber S., El Mokhtari N.E. (2008). Lifelong reduction of LDL-cholesterol related to a common variant in the LDL-receptor gene decreases the risk of coronary artery disease--a Mendelian Randomisation study. PLoS One.

[5] Zakiev E.R., Sukhorukov V.N., Melnichenko A.A., Sobenin I.A., Ivanova E.A., Orekhov A.N. (2016). Lipid composition of circulating multiple-modified low density lipoprotein. Lipids Health Dis..

[6] Tertov V.V., Kaplun V.V., Sobenin I.A., Boytsova E.Y., Bovin N.V., Orekhov A.N. (2001). Human plasma trans-sialidase causes atherogenic modification of low density lipoprotein. Atherosclerosis.

[7] Kunitake S.T., Young S.G., Chen G.C., Pullinger C.R., Zhu S., Pease R.J., Scott J., Hass P., Schilling J., Kane J.P. (1990). Conformation of apolipoprotein B-100 in the low density lipoproteins of tangier disease. Identification of localized conformational response to triglyceride content. J. Biol. Chem..

[8] Wagner A.M., Jorba O., Rigla M., Alonso E., Ordonez-Llanos J., Perez A. (2002). LDL-cholesterol/apolipoprotein B ratio is a good predictor of LDL phenotype B in type 2 diabetes. Acta Diabetol..

[9] Hoogeveen R.C., Gaubatz J.W., Sun W., Dodge R.C., Crosby J.R., Jiang J., Couper D., Virani S.S., Kathiresan S., Boerwinkle E. (2014). Small dense low-density lipoprotein-cholesterol concentrations predict risk for coronary heart disease: The Atherosclerosis Risk In Communities (ARIC) study. Arterioscler Thromb Vasc Biol..

[10] Proctor S.D., Mamo J.C. (2003). Intimal retention of cholesterol derived from apolipoprotein B100- and apolipoprotein B48-containing lipoproteins in carotid arteries of Watanabe heritable hyperlipidemic rabbits. Arterioscler. Thromb. Vasc. Biol..

[11] Mori K., Ishida T., Yasuda T., Monguchi T., Sasaki M., Kondo K., Hasokawa M., Nakajima H., Haraguchi Y., Sun L. (2013). Fasting serum concentration of apolipoprotein B48 represents residual risks in patients with new-onset and chronic coronary artery disease. Clin Chim Acta.

[12] Nakatani K., Sugimoto T., Masuda D., Okano R., Oya T., Monden Y., Yamashita T., Kawase R., Nakaoka H., Inagaki M. (2011). Serum apolipoprotein B-48 levels are correlated with carotid intima-media thickness in subjects with normal serum triglyceride levels. Atherosclerosis.

[13] Nakano T., Nakajima K., Niimi M., Fujita M.Q., Nakajima Y., Takeichi S., Kinoshita M., Matsushima T., Teramoto T., Tanaka A. (2008). Detection of apolipoproteins B-48 and B-100 carrying particles in lipoprotein fractions extracted from human aortic atherosclerotic plaques in sudden cardiac death cases. Clin. Chim. Acta..

[14] Julve J., Martin-Campos J.M., Escola-Gil J.C., Blanco-Vaca F. (2016). Chylomicrons: Advances in biology, pathology, laboratory testing, and therapeutics. Clin Chim Acta..

[15] Barter P.J., Caulfield M., Eriksson M., Grundy S.M., Kastelein J.J., Komajda M., Lopez-Sendon J., Mosca L., Tardif J.C., Waters D.D. (2007). Effects of torcetrapib in patients at high risk for coronary events. N. Engl. J. Med..

[16] Schwartz G.G., Olsson A.G., Abt M., Ballantyne C.M., Barter P.J., Brumm J., Chaitman B.R., Holme I.M., Kallend D., Leiter L.A. (2012). Effects of dalcetrapib in patients with a recent acute coronary syndrome. N. Engl. J. Med..

[17] Rohatgi A., Khera A., Berry J.D., Givens E.G., Ayers C.R., Wedin K.E., Neeland I.J., Yuhanna I.S., Rader D.R., De Lemos J.A. (2014). HDL cholesterol efflux capacity and incident cardiovascular events. N. Engl. J. Med..

[18] Soria-Florido M.T., Castaner O., Lassale C., Estruch R., Salas-Salvado J., Martinez-Gonzalez M.A., Corella D., Ros E., Aros F., Elosua R. (2020). Dysfunctional HDLs are Associated with a Greater Incidence of Acute Coronary Syndrome in a Population at High Cardiovascular Risk: A Nested-Case Control Study. Circulation.

[19] Voight B.F., Peloso G.M., Orho-Melander M., Frikke-Schmidt R., Barbalic M., Jensen M.K., Hindy G., Holm H., Ding E.L., Johnson T. (2012). Plasma HDL cholesterol and risk of myocardial infarction: A mendelian randomisation study. Lancet.

[20] Papageorgiou N., Zacharia E., Androulakis E., Briasoulis A., Charakida M., Tousoulis D. (2016). HDL as a prognostic biomarker for coronary atherosclerosis: The role of inflammation. Expert Opin. Ther. Targets..

[21] Escola-Gil J.C., Calpe-Berdiel L., Palomer X., Ribas V., Ordonez-Llanos J., Blanco-Vaca F. (2006). Antiatherogenic role of high-density lipoproteins: Insights from genetically engineered-mice. Front Biosci.

[22] Mahrooz A., Mackness M., Bagheri A., Ghaffari-Cherati M., Masoumi P. (2019). The epigenetic regulation of paraoxonase 1 (PON1) as an important enzyme in HDL function: The missing link between environmental and genetic regulation. Clin Biochem.

[23] Zalewski A., Macphee C. (2005). Role of lipoprotein-associated phospholipase A2 in atherosclerosis: Biology, epidemiology, and possible therapeutic target. Arterioscler. Thromb. Vasc. Biol..

[24] Vohl M.C., Neville T.A., Kumarathasan R., Braschi S., Sparks D.L. (1999). A novel lecithin-cholesterol acyltransferase antioxidant activity prevents the formation of oxidized lipids during lipoprotein oxidation. Biochemistry.

[25] Loscalzo J. (2001). Nitric oxide insufficiency, platelet activation, and arterial thrombosis. Circ. Res..

[26] Besler C., Luscher T.F., Landmesser U. (2012). Molecular mechanisms of vascular effects of High-density lipoprotein: Alterations in cardiovascular disease. EMBO Mol. Med..

[27] Nofer J.R., Van der Giet M., Tolle M., Wolinska I., Von Wnuck Lipinski K., Baba H.A., Tietge U.J., Godecke A., Ishii I., Kleuser B. (2004). HDL induces NO-dependent vasorelaxation via the lysophospholipid receptor S1P3. J. Clin. Investig..

[28] Freedman J.E., Loscalzo J. (2003). Nitric oxide and its relationship to thrombotic disorders. J. Thromb. Haemost..

[29] Calkin A.C., Drew B.G., Ono A., Duffy S.J., Gordon M.V., Schoenwaelder S.M., Sviridov D., Cooper M.E., Kingwell B.A., Jackson S.P. (2009). Reconstituted high-density lipoprotein attenuates platelet function in individuals with type 2 diabetes mellitus by promoting cholesterol efflux. Circulation.

[30] Lerch P.G., Spycher M.O., Doran J.E. (1998). Reconstituted high density lipoprotein (rHDL) modulates platelet activity in vitro and ex vivo. Thromb. Haemost..

[31] Drew B.G., Duffy S.J., Formosa M.F., Natoli A.K., Henstridge D.C., Penfold S.A., Thomas W.G., Mukhamedova N., De Courten B., Forbes J.M. (2009). High-density lipoprotein modulates glucose metabolism in patients with type 2 diabetes mellitus. Circulation.

[32] Wu A., Hinds C.J., Thiemermann C. (2004). High-density lipoproteins in sepsis and septic shock: Metabolism, actions, and therapeutic applications. Shock.

[33] Levine D.M., Parker T.S., Donnelly T.M., Walsh A., Rubin A.L. (1993). In vivo protection against endotoxin by plasma high density lipoprotein. Proc. Natl. Acad. Sci. USA.

[34] Rosenson R.S., Brewer H.B., Ansell B.J., Barter P., Chapman M.J., Heinecke J.W., Kontush A., Tall A.R., Webb N.R. (2016). Dysfunctional HDL and atherosclerotic cardiovascular disease. Nat Rev Cardiol.

[35] Vaisar T., Tang C., Babenko I., Hutchins P., Wimberger J., Suffredini A.F., Heinecke J.W. (2015). Inflammatory remodeling of the HDL proteome impairs cholesterol efflux capacity. J Lipid Res.

[36] Han C.Y., Tang C., Guevara M.E., Wei H., Wietecha T., Shao B., Subramanian S., Omer M., Wang S., O’Brien K.D. (2016). Serum amyloid A impairs the antiinflammatory properties of HDL. J Clin Invest.

[37] Van Lenten B.J., Wagner A.C., Nayak D.P., Hama S., Navab M., Fogelman A.M. (2001). High-density lipoprotein loses its anti-inflammatory properties during acute influenza a infection. Circulation.

[38] Miller N.E. (1987). Associations of high-density lipoprotein subclasses and apolipoproteins with ischemic heart disease and coronary atherosclerosis. Am. Heart J..

[39] Bakogianni M.C., Kalofoutis C.A., Skenderi K.I., Kalofoutis A.T. (2001). Clinical evaluation of plasma high-density lipoprotein subfractions (HDL2, HDL3) in non-insulin-dependent diabetics with coronary artery disease. J. Diabetes Complicat..

[40] Freedman D.S., Otvos J.D., Jeyarajah E.J., Barboriak J.J., Anderson A.J., Walker J.A. (1998). Relation of lipoprotein subclasses as measured by proton nuclear magnetic resonance spectroscopy to coronary artery disease. Arterioscler. Thromb. Vasc. Biol..

[41] Zeljkovic A., Vekic J., Spasojevic-Kalimanovska V., Jelic-Ivanovic Z., Bogavac-Stanojevic N., Gulan B., Spasic S. (2010). LDL and HDL subclasses in acute ischemic stroke: Prediction of risk and short-term mortality. Atherosclerosis.

[42] Mowat B.F., Skinner E.R., Wilson H.M., Leng G.C., Fowkes F.G., Horrobin D. (1997). Alterations in plasma lipids, lipoproteins and high density lipoprotein subfractions in peripheral arterial disease. Atherosclerosis.

[43] Borggreve S.E., De Vries R., Dullaart R.P. (2003). Alterations in high-density lipoprotein metabolism and reverse cholesterol transport in insulin resistance and type 2 diabetes mellitus: Role of lipolytic enzymes, lecithin:Cholesterol acyltransferase and lipid transfer proteins. Eur. J. Clin. Investig..

[44] Soderlund S., Soro-Paavonen A., Ehnholm C., Jauhiainen M., Taskinen M.R. (2005). Hypertriglyceridemia is associated with prebeta-HDL concentrations in subjects with familial low HDL. J. Lipid Res..

[45] De Beer M.C., Durbin D.M., Cai L., Jonas A., De Beer F.C., Van der Westhuyzen D.R. (2001). Apolipoprotein A-I conformation markedly influences HDL interaction with scavenger receptor BI. J. Lipid Res..

[46] Thuahnai S.T., Lund-Katz S., Dhanasekaran P., De la Llera-Moya M., Connelly M.A., Williams D.L., Rothblat G.H., Phillips M.C. (2004). Scavenger receptor class B type I-mediated cholesteryl ester-selective uptake and efflux of unesterified cholesterol. Influence of high density lipoprotein size and structure. J. Biol. Chem..

[47] Sparks D.L., Davidson W.S., Lund-Katz S., Phillips M.C. (1995). Effects of the neutral lipid content of high density lipoprotein on apolipoprotein A-I structure and particle stability. J. Biol. Chem..

[48] Phillips M.C., Johnson W.J., Rothblat G.H. (1987). Mechanisms and consequences of cellular cholesterol exchange and transfer. Biochim. Biophys. Acta..

[49] Hernaez A., Fernandez-Castillejo S., Farras M., Catalan U., Subirana I., Montes R., Sola R., Munoz-Aguayo D., Gelabert-Gorgues A., Diaz-Gil O. (2014). Olive oil polyphenols enhance high-density lipoprotein function in humans: A randomized controlled trial. Arterioscler. Thromb. Vasc. Biol..

[50] Covas M.I., Nyyssonen K., Poulsen H.E., Kaikkonen J., Zunft H.J., Kiesewetter H., Gaddi A., De la Torre R., Mursu J., Baumler H. (2006). The effect of polyphenols in olive oil on heart disease risk factors: A randomized trial. Ann. Intern. Med..

[51] Fito M., Estruch R., Salas-Salvado J., Martinez-Gonzalez M.A., Aros F., Vila J., Corella D., Diaz O., Saez G., De la Torre R. (2014). Effect of the Mediterranean diet on heart failure biomarkers: A randomized sample from the PREDIMED trial. Eur. J. Heart Fail..

[52] Martinez-Gonzalez M.A., Ros E., Estruch R. (2018). Primary Prevention of Cardiovascular Disease with a Mediterranean Diet Supplemented with Extra-Virgin Olive Oil or Nuts. N. Engl. J. Med..

[53] Donini L.M., Serra-Majem L., Bullo M., Gil A., Salas-Salvado J. (2015). The Mediterranean diet: Culture, health and science. Br. J. Nutr..

[54] Owen R.W., Mier W., Giacosa A., Hull W.E., Spiegelhalder B., Bartsch H. (2000). Phenolic compounds and squalene in olive oils: The concentration and antioxidant potential of total phenols, simple phenols, secoiridoids, lignansand squalene. Food Chem. Toxicol. Int. J. Publ. Br. Ind. Biol. Res. Assoc..

[55] EFSA Panel on Dietetic Products (2011). Nutrition and allergies, N. The Effect of Polyphenols in Olive Oil on Heart Diseases. EFSA J..

[56] Neuzil J., Thomas S.R., Stocker R. (1997). Requirement for, promotion, or inhibition by alpha-tocopherol of radical-induced initiation of plasma lipoprotein lipid peroxidation. Free Radic. Biol. Med..

[57] Wilson T., Knight T.J., Beitz D.C., Lewis D.S., Engen R.L. (1996). Resveratrol promotes atherosclerosis in hypercholesterolemic rabbits. Life Sci..

[58] Acin S., Navarro M.A., Arbones-Mainar J.M., Guillen N., Sarria A.J., Carnicer R., Surra J.C., Orman I., Segovia J.C., Torre Rde L. (2006). Hydroxytyrosol administration enhances atherosclerotic lesion development in apo E deficient mice. J. Biochem..

[59] Steffen B.T., Guan W., Remaley A.T., Paramsothy P., Heckbert S.R., McClelland R.L., Greenland P., Michos E.D., Tsai M.Y. (2015). Use of lipoprotein particle measures for assessing coronary heart disease risk post-American Heart Association/American College of Cardiology guidelines: The Multi-Ethnic Study of Atherosclerosis. Arterioscler. Thromb. Vasc. Biol..

[60] Hernaez A., Castaner O., Goday A., Ros E., Pinto X., Estruch R., Salas-Salvado J., Corella D., Aros F., Serra-Majem L. (2017). The Mediterranean Diet decreases LDL atherogenicity in high cardiovascular risk individuals: A randomized controlled trial. Mol. Nutr. Food Res..

[61] Hernaez A., Remaley A.T., Farras M., Fernandez-Castillejo S., Subirana I., Schroder H., Fernandez-Mampel M., Munoz-Aguayo D., Sampson M., Sola R. (2015). Olive Oil Polyphenols Decrease LDL Concentrations and LDL Atherogenicity in Men in a Randomized Controlled Trial. J. Nutr..

[62] Cherki M., Derouiche A., Drissi A., El Messal M., Bamou Y., Idrissi-Ouadghiri A., Khalil A., Adlouni A. (2005). Consumption of argan oil may have an antiatherogenic effect by improving paraoxonase activities and antioxidant status: Intervention study in healthy men. Nutr. Metab. Cardiovasc. Dis..

[63] Fito M., Cladellas M., De la Torre R., Marti J., Alcantara M., Pujadas-Bastardes M., Marrugat J., Bruguera J., Lopez-Sabater M.C., Vila J. (2005). Antioxidant effect of virgin olive oil in patients with stable coronary heart disease: A randomized, crossover, controlled, clinical trial. Atherosclerosis.

[64] Peroulis N., Androutsopoulos V.P., Notas G., Koinaki S., Giakoumaki E., Spyros A., Manolopoulou E., Kargaki S., Tzardi M., Moustou E. (2019). Significant metabolic improvement by a water extract of olives: Animal and human evidence. Eur. J. Nutr..

[65] Fernandez-Castillejo S., Valls R.M., Castaner O., Rubio L., Catalan U., Pedret A., Macia A., Sampson M.L., Covas M.I., Fito M. (2016). Polyphenol rich olive oils improve lipoprotein particle atherogenic ratios and subclasses profile: A randomized, crossover, controlled trial. Nutr. Food Res..

[66] Martin-Pelaez S., Mosele J.I., Pizarro N., Farras M., De la Torre R., Subirana I., Perez-Cano F.J., Castaner O., Sola R., Fernandez-Castillejo S. (2017). Effect of virgin olive oil and thyme phenolic compounds on blood lipid profile: Implications of human gut microbiota. Eur. J. Nutr..

[67] Weinbrenner T., Fito M., De la Torre R., Saez G.T., Rijken P., Tormos C., Coolen S., Albaladejo M.F., Abanades S., Schroder H. (2004). Olive oils high in phenolic compounds modulate oxidative/antioxidative status in men. J. Nutr..

[68] Farras M., Valls R.M., Fernandez-Castillejo S., Giralt M., Sola R., Subirana I., Motilva M.J., Konstantinidou V., Covas M.I., Fito M. (2013). Olive oil polyphenols enhance the expression of cholesterol efflux related genes in vivo in humans. A randomized controlled trial. J. Nutr. Biochem..

[69] Perrone M.A., Gualtieri P., Gratteri S., Ali W., Sergi D., Muscoli S., Cammarano A., Bernardini S., Di Renzo L., Romeo F. (2019). Effects of postprandial hydroxytyrosol and derivates on oxidation of LDL, cardiometabolic state and gene expression: A nutrigenomic approach for cardiovascular prevention. J. Cardiovasc. Med..

[70] Konstantinidou V., Khymenets O., Covas M.I., De la Torre R., Munoz-Aguayo D., Anglada R., Farre M., Fito M. (2009). Time course of changes in the expression of insulin sensitivity-related genes after an acute load of virgin olive oil. Omics J. Integr. Biol..

[71] Valls R.M., Pedret A., Fernández-Castillejo S., Catalán Ú., Romeu M., Giralt M., Sáez G.T., Fitó M., De la Torre R., Covas M.I. (2017). Virgin olive oil enriched with its own phenolic compounds or complemented with thyme improves endothelial function: The potential role of plasmatic fat-soluble vitamins. A double blind, randomized, controlled, cross-over clinical trial. J. Funct. Foods..

[72] Ebenbichler C.F., Kirchmair R., Egger C., Patsch J.R. (1995). Postprandial state and atherosclerosis. Curr. Opin. Lipidol..

[73] Roche H.M., Gibney M.J. (2000). The impact of postprandial lipemia in accelerating atherothrombosis. J. Cardiovasc. Risk..

[74] Cullen P. (2003). Triacylglycerol-rich lipoproteins and atherosclerosis--where is the link?. Biochem. Soc. Trans..

[75] Groot P.H., Van Stiphout W.A., Krauss X.H., Jansen H., Van Tol A., Van Ramshorst E., Chin-On S., Hofman A., Cresswell S.R., Havekes L. (1991). Postprandial lipoprotein metabolism in normolipidemic men with and without coronary artery disease. Arterioscler. Thromb. J. Vasc. Biol..

[76] Gomez-Marin B., Gomez-Delgado F., Lopez-Moreno J., Alcala-Diaz J.F., Jimenez-Lucena R., Torres-Pena J.D., Garcia-Rios A., Ortiz-Morales A.M., Yubero-Serrano E.M., Del Mar Malagon M. (2018). Long-term consumption of a Mediterranean diet improves postprandial lipemia in patients with type 2 diabetes: The Cordioprev randomized trial. Am. J. Clin. Nutr..

[77] Thomsen C., Storm H., Holst J.J., Hermansen K. (2003). Differential effects of saturated and monounsaturated fats on postprandial lipemia and glucagon-like peptide 1 responses in patients with type 2 diabetes. Am. J. Clin. Nutr..

[78] Mekki N., Charbonnier M., Borel P., Leonardi J., Juhel C., Portugal H., Lairon D. (2002). Butter differs from olive oil and sunflower oil in its effects on postprandial lipemia and triacylglycerol-rich lipoproteins after single mixed meals in healthy young men. J. Nutr..

[79] Rodríguez-Villar C., Casals E., Pérez-Heras A., Zambón D., Gomis R., Ros E. (2000). High–Monounsaturated Fat, Olive Oil–Rich Diet Has Effects Similar to a High-Carbohydrate Diet on Fasting and Postprandial State and Metabolic Profiles of Patients With Type 2 Diabetes. Metab. Clin. Exp..

[80] Abia R., Pacheco Y.M., Perona J.S., Montero E., Muriana F.J., Ruiz-Gutierrez V. (2001). The metabolic availability of dietary triacylglycerols from two high oleic oils during the postprandial period does not depend on the amount of oleic acid ingested by healthy men. J. Nutr..

[81] Perona J.S., Martinez-Gonzalez J., Sanchez-Dominguez J.M., Badimon L., Ruiz-Gutierrez V. (2004). The unsaponifiable fraction of virgin olive oil in chylomicrons from men improves the balance between vasoprotective and prothrombotic factors released by endothelial cells. J. Nutr..

[82] Cabello-Moruno R., Perona J.S., Osada J., Garcia M., Ruiz-Gutierrez V. (2007). Modifications in postprandial triglyceride-rich lipoprotein composition and size after the intake of pomace olive oil. J. Am. Coll. Nutr..

[83] Weintraub M.S., Zechner R., Brown A., Eisenberg S., Breslow J.L. (1988). Dietary polyunsaturated fats of the W-6 and W-3 series reduce postprandial lipoprotein levels. Chronic and acute effects of fat saturation on postprandial lipoprotein metabolism. J. Clin. Investig..

[84] Sakr S.W., Attia N., Haourigui M., Paul J.L., Soni T., Vacher D., Girard-Globa A. (1997). Fatty acid composition of an oral load affects chylomicron size in human subjects. Br. J. Nutr..

[85] Rahman M.H., Avella M.A., Botham K.M. (2000). The fatty acid composition of chylomicrons influences the rate of their lipolysis in vivo. Nutr. Metab. Cardiovasc. Dis. NMCD.

[86] Jackson K.G., Robertson M.D., Fielding B.A., Frayn K.N., Williams C.M. (2002). Olive oil increases the number of triacylglycerol-rich chylomicron particles compared with other oils: An effect retained when a second standard meal is fed. Am. J. Clin. Nutr..

[87] Abia R., Pacheco Y.M., Montero E., Ruiz-Gutierrez V., Muriana F.J. (2003). Distribution of fatty acids from dietary oils into phospholipid classes of triacylglycerol-rich lipoproteins in healthy subjects. Life Sci..

[88] Lambert M.S., Avella M.A., Berhane Y., Shervill E., Botham K.M. (2001). The fatty acid composition of chylomicron remnants influences their binding and internalization by isolated hepatocytes. Eur. J. Biochem..

[89] Perona J.S., Avella M., Botham K.M., Ruiz-Gutierrez V. (2006). Uptake of triacylglycerol-rich lipoproteins of differing triacylglycerol molecular species and unsaponifiable content by liver cells. Br. J. Nutr..

[90] Lopez-Soldado I., Avella M., Botham K.M. (2009). Differential influence of different dietary fatty acids on very low-density lipoprotein secretion when delivered to hepatocytes in chylomicron remnants. Metab. Clin. Exp..

[91] Perez-Martinez P., Garcia-Quintana J.M., Yubero-Serrano E.M., Tasset-Cuevas I., Tunez I., Garcia-Rios A., Delgado-Lista J., Marin C., Perez-Jimenez F., Roche H.M. (2010). Postprandial oxidative stress is modified by dietary fat: Evidence from a human intervention study. Clin. Sci..

[92] Bellido C., Lopez-Miranda J., Blanco-Colio L.M., Perez-Martinez P., Muriana F.J., Martin-Ventura J.L., Marin C., Gomez P., Fuentes F., Egido J. (2004). Butter and walnuts, but not olive oil, elicit postprandial activation of nuclear transcription factor kappaB in peripheral blood mononuclear cells from healthy men. Am. J. Clin. Nutr..

[93] Perez-Herrera A., Delgado-Lista J., Torres-Sanchez L.A., Rangel-Zuniga O.A., Camargo A., Moreno-Navarrete J.M., Garcia-Olid B., Quintana-Navarro G.M., Alcala-Diaz J.F., Munoz-Lopez C. (2012). The postprandial inflammatory response after ingestion of heated oils in obese persons is reduced by the presence of phenol compounds. Mol. Nutr. Food Res..

[94] Pacheco Y.M., Abia R., Perona J.S., Reina M., Ruiz-Gutierrez V., Montero E., Muriana F.J. (2001). Triacylglycerol-rich lipoproteins interact with human vascular cells in a lipid-dependent fashion. J. Agric. Food Chem..

[95] De Pascale C., Avella M., Perona J.S., Ruiz-Gutierrez V., Wheeler-Jones C.P., Botham K.M. (2006). Fatty acid composition of chylomicron remnant-like particles influences their uptake and induction of lipid accumulation in macrophages. FEBS J..

[96] Quintero-Florez A., Sinausia Nieva L., Sanchez-Ortiz A., Beltran G., Perona J.S. (2015). The Fatty Acid Composition of Virgin Olive Oil from Different Cultivars Is Determinant for Foam Cell Formation by Macrophages. J. Agric. Food Chem..

[97] Derouiche A., Cherki M., Drissi A., Bamou Y., El Messal M., Idrissi-Oudghiri A., Lecerf J.M., Adlouni A. (2005). Nutritional intervention study with argan oil in man: Effects on lipids and apolipoproteins. Ann. Nutr. Metab..

[98] Pedret A., Catalan U., Fernandez-Castillejo S., Farras M., Valls R.M., Rubio L., Canela N., Aragones G., Romeu M., Castaner O. (2015). Impact of Virgin Olive Oil and Phenol-Enriched Virgin Olive Oils on the HDL Proteome in Hypercholesterolemic Subjects: A Double Blind, Randomized, Controlled, Cross-Over Clinical Trial (VOHF Study). PloS one.

[99] Arbones-Mainar J.M., Navarro M.A., Carnicer R., Guillen N., Surra J.C., Acin S., Guzman M.A., Sarria A.J., Arnal C., Aguilera M.P. (2007). Accelerated atherosclerosis in apolipoprotein E-deficient mice fed Western diets containing palm oil compared with extra virgin olive oils: A role for small, dense high-density lipoproteins. Atherosclerosis.

[100] Sola R., Fito M., Estruch R., Salas-Salvado J., Corella D., De La Torre R., Munoz M.A., Lopez-Sabater Mdel C., Martinez-Gonzalez M.A., Aros F. (2011). Effect of a traditional Mediterranean diet on apolipoproteins B, A-I, and their ratio: A randomized, controlled trial. Atherosclerosis.

[101] Lopez-Miranda J., Gomez P., Castro P., Marin C., Paz E., Bravo M.D., Blanco J., Jimenez-Pereperez J., Fuentes F., Perez-Jimenez F. (2000). Mediterranean diet improves low density lipoprotein susceptibility to oxidative modifications. Med. Clin..

[102] Helal O., Berrougui H., Loued S., Khalil A. (2013). Extra-virgin olive oil consumption improves the capacity of HDL to mediate cholesterol efflux and increases ABCA1 and ABCG1 expression in human macrophages. Br. J. Nutr..

[103] Damasceno N.R., Sala-Vila A., Cofan M., Perez-Heras A.M., Fito M., Ruiz-Gutierrez V., Martinez-Gonzalez M.A., Corella D., Aros F., Estruch R. (2013). Mediterranean diet supplemented with nuts reduces waist circumference and shifts lipoprotein subfractions to a less atherogenic pattern in subjects at high cardiovascular risk. Atherosclerosis.

[104] Mangas-Cruz M.A., Fernandez-Moyano A., Albi T., Guinda A., Relimpio F., Lanzon A., Pereira J.L., Serrera J.L., Montilla C., Astorga R. (2001). Effects of minor constituents (non-glyceride compounds) of virgin olive oil on plasma lipid concentrations in male Wistar rats. Clin. Nutr..

[105] Farras M., Castaner O., Martin-Pelaez S., Hernaez A., Schroder H., Subirana I., Munoz-Aguayo D., Gaixas S., Torre Rde L., Farre M. (2015). Complementary phenol-enriched olive oil improves HDL characteristics in hypercholesterolemic subjects. A randomized, double-blind, crossover, controlled trial. The VOHF study. Mol. Nutr. Food Res..

[106] Singer P., Jaeger W., Berger I., Barleben H., Wirth M., Richter-Heinrich E., Voigt S., Godicke W. (1990). Effects of dietary oleic, linoleic and alpha-linolenic acids on blood pressure, serum lipids, lipoproteins and the formation of eicosanoid precursors in patients with mild essential hypertension. J. Hum. Hypertens..

[107] Van Tol A., Terpstra A.H., Van den Berg P., Beynen A.C. (1999). Dietary corn oil versus olive oil enhances HDL protein turnover and lowers HDL cholesterol levels in hamsters. Atherosclerosis.

[108] Terpstra A.H., Van den Berg P., Jansen H., Beynen A.C., Van Tol A. (2000). Decreasing dietary fat saturation lowers HDL-cholesterol and increases hepatic HDL binding in hamsters. Br. J. Nutr..

[109] Martinez-Beamonte R., Navarro M.A., Acin S., Guillen N., Barranquero C., Arnal C., Surra J., Osada J. (2013). Postprandial changes in high density lipoproteins in rats subjected to gavage administration of virgin olive oil. PLoS ONE.

[110] Gabas-Rivera C., Barranquero C., Martinez-Beamonte R., Navarro M.A., Surra J.C., Osada J. (2014). Dietary squalene increases high density lipoprotein-cholesterol and paraoxonase 1 and decreases oxidative stress in mice. PloS one.

[111] Khera A.V., Cuchel M., De la Llera-Moya M., Rodrigues A., Burke M.F., Jafri K., French B.C., Phillips J.A., Mucksavage M.L., Wilensky R.L. (2011). Cholesterol efflux capacity, high-density lipoprotein function, and atherosclerosis. N. Engl. J. Med..

[112] Li X.M., Tang W.H., Mosior M.K., Huang Y., Wu Y., Matter W., Gao V., Schmitt D., Didonato J.A., Fisher E.A. (2013). Paradoxical association of enhanced cholesterol efflux with increased incident cardiovascular risks. Arterioscler. Thromb. Vasc. Biol..

[113] Fernandez-Castillejo S., Rubio L., Hernaez A., Catalan U., Pedret A., Valls R.M., Mosele J.I., Covas M.I., Remaley A.T., Castaner O. (2017). Determinants of HDL Cholesterol Efflux Capacity after Virgin Olive Oil Ingestion: Interrelationships with Fluidity of HDL Monolayer. Mol. Nutr. Food Res..

[114] Hernaez A., Castaner O., Elosua R., Pinto X., Estruch R., Salas-Salvado J., Corella D., Aros F., Serra-Majem L., Fiol M. (2017). Mediterranean Diet Improves High-Density Lipoprotein Function in High-Cardiovascular-Risk Individuals: A Randomized Controlled Trial. Circulation.

[115] Hernaez A., Sanllorente A., Castaner O., Martinez-Gonzalez M.A., Ros E., Pinto X., Estruch R., Salas-Salvado J., Corella D., Alonso-Gomez A.M. (2019). Increased Consumption of Virgin Olive Oil, Nuts, Legumes, Whole Grains, and Fish Promotes HDL Functions in Humans. Mol. Nutr. Food Res..

[116] Hao J., Shen W., Yu G., Jia H., Li X., Feng Z., Wang Y., Weber P., Wertz K., Sharman E. (2010). Hydroxytyrosol promotes mitochondrial biogenesis and mitochondrial function in 3T3-L1 adipocytes. J. Nutr. Biochem..

[117] Konstantinidou V., Covas M.I., Munoz-Aguayo D., Khymenets O., De la Torre R., Saez G., Tormos Mdel C., Toledo E., Marti A., Ruiz-Gutierrez V. (2010). In vivo nutrigenomic effects of virgin olive oil polyphenols within the frame of the Mediterranean diet: A randomized controlled trial. J. Off. Publ. Fed. Am. Soc. Exp. Biol..

[118] Ross R. (1999). Atherosclerosis--an inflammatory disease. N. Engl. J. Med..

[119] Kontush A., Chapman M.J. (2006). Functionally defective high-density lipoprotein: A new therapeutic target at the crossroads of dyslipidemia, inflammation, and atherosclerosis. Pharmacol. Rev..

[120] Barter P.J., Nicholls S., Rye K.A., Anantharamaiah G.M., Navab M., Fogelman A.M. (2004). Antiinflammatory properties of HDL. Circ. Res..

[121] Bursill C.A., Castro M.L., Beattie D.T., Nakhla S., Van der Vorst E., Heather A.K., Barter P.J., Rye K.A. (2010). High-density lipoproteins suppress chemokines and chemokine receptors in vitro and in vivo. Arterioscler. Thromb. Vasc. Biol..

[122] Murphy A.J., Woollard K.J., Suhartoyo A., Stirzaker R.A., Shaw J., Sviridov D., Chin-Dusting J.P. (2011). Neutrophil activation is attenuated by high-density lipoprotein and apolipoprotein A-I in in vitro and in vivo models of inflammation. Arterioscler. Thromb. Vasc. Biol..

[123] Yvan-Charvet L., Welch C., Pagler T.A., Ranalletta M., Lamkanfi M., Han S., Ishibashi M., Li R., Wang N., Tall A.R. (2008). Increased inflammatory gene expression in ABC transporter-deficient macrophages: Free cholesterol accumulation, increased signaling via toll-like receptors, and neutrophil infiltration of atherosclerotic lesions. Circulation.

[124] Valls R.M., Farras M., Suarez M., Fernandez-Castillejo S., Fito M., Konstantinidou V., Fuentes F., Lopez-Miranda J., Giralt M., Covas M.I. (2015). Effects of functional olive oil enriched with its own phenolic compounds on endothelial function in hypertensive patients. A randomised controlled trial. Food Chem..

[125] Moreno-Luna R., Munoz-Hernandez R., Miranda M.L., Costa A.F., Jimenez-Jimenez L., Vallejo-Vaz A.J., Muriana F.J., Villar J., Stiefel P. (2012). Olive oil polyphenols decrease blood pressure and improve endothelial function in young women with mild hypertension. Am. J. Hypertens..

[126] Schwingshackl L., Hoffmann G. (2014). Mediterranean dietary pattern, inflammation and endothelial function: A systematic review and meta-analysis of intervention trials. Nutr. Metab. Cardiovasc. Dis..

[127] Loued S., Berrougui H., Componova P., Ikhlef S., Helal O., Khalil A. (2013). Extra-virgin olive oil consumption reduces the age-related decrease in HDL and paraoxonase 1 anti-inflammatory activities. Br. J. Nutr..

[128] Richard C., Couture P., Desroches S., Nehme B., Bourassa S., Droit A., Lamarche B. (2014). Effect of an isoenergetic traditional Mediterranean diet on the high-density lipoprotein proteome in men with the metabolic syndrome. J. Nutr. Nutr..

[129] Meza-Miranda E.R., Rangel-Zuniga O.A., Marin C., Perez-Martinez P., Delgado-Lista J., Haro C., Pena-Orihuela P., Jimenez-Morales A.I., Malagon M.M., Tinahones F.J. (2016). Virgin olive oil rich in phenolic compounds modulates the expression of atherosclerosis-related genes in vascular endothelium. Eur. J. Nutr..

